# Quinolones Modulate Ghrelin Receptor Signaling: Potential for a Novel Small Molecule Scaffold in the Treatment of Cachexia

**DOI:** 10.3390/ijms19061605

**Published:** 2018-05-30

**Authors:** Cristina Torres-Fuentes, Elena Pastor-Cavada, Rafael Cano, Dalia Kandil, Rachel Shanahan, Rocio Juan, Hamdy Shaban, Gerard P. McGlacken, Harriët Schellekens

**Affiliations:** 1 APC Microbiome Ireland, University College Cork, T12 YT20 Cork, Ireland; ctorresfuentes@ucdavis.edu (C.T.-F.); elenapastorcavada@gmail.com (E.P.-C.); 2 School of Chemistry and the Analytical and Biological Chemistry Research Facility (ABCRF), University College Cork, T12 YT20 Cork, Ireland; rafael.canom@uam.es (R.C.); rachelshanahan91@gmail.com (R.S.); g.mcglacken@ucc.ie (G.P.M.); 3 Food for Health Ireland, University College Cork, T12 YT20 Cork, Ireland; dkandil@ucc.ie (D.K.); hamdy.shaban@gmx.ch (H.S.); 4 Department of Anatomy and Neuroscience, University College Cork, T12 YT20 Cork, Ireland; 5 Plant Biology and Ecology Department, Seville University, 41012 Seville, Spain; rjuan@us.es

**Keywords:** ghrelin, quinolones, cachexia, GHS-R1a

## Abstract

Cachexia is a metabolic wasting disorder characterized by progressive weight loss, muscle atrophy, fatigue, weakness, and appetite loss. Cachexia is associated with almost all major chronic illnesses including cancer, heart failure, obstructive pulmonary disease, and kidney disease and significantly impedes treatment outcome and therapy tolerance, reducing physical function and increasing mortality. Current cachexia treatments are limited and new pharmacological strategies are needed. Agonists for the growth hormone secretagogue (GHS-R1a), or ghrelin receptor, prospectively regulate the central regulation of appetite and growth hormone secretion, and therefore have tremendous potential as cachexia therapeutics. Non-peptide GHS-R1a agonists are of particular interest, especially given the high gastrointestinal degradation of peptide-based structures, including that of the endogenous ligand, ghrelin, which has a half-life of only 30 min. However, few compounds have been reported in the literature as non-peptide GHS-R1a agonists. In this paper, we investigate the in vitro potential of quinolone compounds to modulate the GHS-R1a in both transfected human cells and mouse hypothalamic cells. These chemically synthesized compounds demonstrate a promising potential as GHS-R1a agonists, shown by an increased intracellular calcium influx. Further studies are now warranted to substantiate and exploit the potential of these novel quinolone-based compounds as orexigenic therapeutics in conditions of cachexia and other metabolic and eating disorders.

## 1. Introduction

Cachexia is an advancing metabolic disorder that leads to substantial weight and muscle loss and affects over nine million people worldwide [1]. Cachexia is associated with the late stages of many major chronic illnesses such as cancer, heart failure, chronic obstructive pulmonary disease, and kidney disease [2]. For many years, physicians and researchers have focused their attention on the primary illness and it took until 2006 for a formal definition for cachexia to emerge [3]. It has been defined as a “complex metabolic syndrome associated with underlying illness and characterized by loss of muscle with or without loss of fat mass” [4]. More recently, cachexia is further defined as weight loss greater than 5% of body weight over 12 months or less, in the presence of chronic illness, or a body mass index (BMI) lower than 20 kg/m^2^ [5]. The latest research suggests that cachexia is more than a muscle disease and is related to problems in the brain’s regulation of appetite and feeding and to inflammation and metabolic imbalances [1]. 

Current cachexia treatments are limited and new pharmacological strategies are urgently needed [6]. Agonists of the ghrelin receptor (GHS-R1a) have recently received attention for cachexia treatment. Ghrelin, which is the endogenous GHS-R1a ligand, is so far the only known peripherally-derived hormone with orexigenic effects [7]. However, ghrelin has several other unique functions in the regulation of energy homeostasis [8,9,10] and is involved in the modulation of different physiological activities in the organism [10,11]. For example, ghrelin has been shown to be involved in the central regulation of muscle metabolism [12]. As a 28-amino acid peptide hormone, it is primarily produced in the oxyntic mucosa of the stomach as well as in other gastrointestinal tissues [13]. It acts as the endogenous ligand for GHS-R1a and causes the release of a growth hormone from the pituitary gland [14]. Ghrelin administration increases food intake by activating GHS-R1a located in neurons of the arcuate nucleus of the hypothalamus [10,15,16] and stimulates gastrointestinal motility while decreasing nausea and vomiting [17]. Ghrelin has a central role in regulating the energy balance by decreasing energy consumption via the inhibition of the sympathetic nervous system output to brown adipose tissue, which reduces thermogenesis [18]. Therefore, activation of ghrelin signaling may promote a positive energy balance, increased food intake, and body weight gain [19]. However, ghrelin administration efficacy in patients is limited due to the need for parenteral administration and a short half-life (30 min) [20]. Therefore, the development of new GHS-R1a agonists with a longer half-life allows for once-daily administration, which could be of great potential benefit in the treatment of cachexia [21]. Given the problematic degradation of peptidic based structures [22,23], non-peptide GHS-R1a agonists may have higher potential as therapeutics. However, perhaps surprisingly, very few compounds have been reported in the literature as promising non-peptide GHS-R1a agonists. Anamorelin, (Helsinn Healthcare SA, Lugano, Switzerland), which is a non-peptide, orally-active compound, is the only centrally-penetrant compound described to date. It is shown to have appetite-enhancing and anabolic effects via selective agonism of the GHS-R1a. Anamorelin is currently under development for the treatment of cancer cachexia [24,25,26,27,28]. Other compounds such as the polyphenol naringin, the gamma-secretase inhibitor “semagacestat,” and biosynthetic intermediates of teaghrelins and emoghrelin have also shown a potential as GHS-R1a agonists [29,30,31]. Recently, we also demonstrated a novel, non-peptidic, 2-pyridone bears a crucial trifluoromethyl group [32,33] at the 3-position, which demonstrated a selective GHS-R1a agonist potential in vitro and promised modulation of food intake in vivo [22]. However, further studies are warranted to identify novel selective GHS-R1a-targeting small molecule agonists with increased potency and selectivity as well as improved pharmacokinetics and bioavailability. New and improved scaffolds are required to allow a greater diversification of the molecular structure. In this regard, quinolone compounds (which are considered privileged molecular scaffolds) could meet some of these requirements.

Quinolone compounds have a very diverse biological profile which includes widespread, medicinally interesting properties ranging from antiallergenic and anticancer to antimicrobial activities [34]. Additionally, quinolones have been used in the development of numerous synthetic drugs [34]. The success of the quinolone moiety across numerous areas of biological importance is underpinned by the ability to selectively modify its molecular framework and, therefore, grant favorable biological properties. For example, recently, quinolones have been studied as signaling molecules for the interruption of quorum sensing in *Pseudomonas aeruginosa* and other pathogens [35,36,37,38,39]. The interactions have shown that exquisite specificity-of-structure is often required for biological activity, which renders the easily-decorated quinolones as an excellent molecular base-point. Therefore, the aim of this study was to investigate the ability of chemically synthesized quinolone compounds to modulate the GHS-R1a knowing that the molecular structures could be easily modified and tailored.

## 2. Results

### 2.1. Quinolones Compounds and Their Effects on GHS-R1a Signaling

#### 2.1.1. Quinolones Synthesis

**Figure 1:**
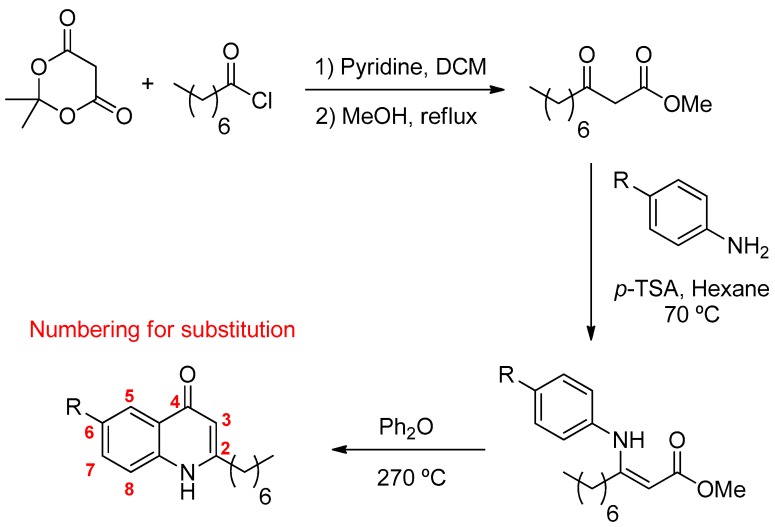
Quinolones synthesis. The β-keto-ester, methyl-3-oxodecanoate was prepared by acylation of Meldrum’s acid (2,2-dimethyl-1,3-dioxane-4,6-dione) with octanoyl chloride, which was followed by methanolysis. Condensation of the β-keto-ester with the corresponding aniline in the presence of p-toluene sulfonic acid using a Dean-Stark apparatus and subsequent Conrad-Limpach cyclisation of the enamine at reflux in diphenyl ether gave the corresponding 2-heptyl-4-quinolone.

The 4-Hydroxy-2-heptylquinoline and related analogs (see Figure 1) were prepared using a procedure involving a Conrad-Limpach type cyclisation. The β-keto-ester, methyl-3-oxodecanoate was prepared by acylation of Meldrum’s acid (2,2-dimethyl-1,3-dioxane-4,6-dione) with octanoyl chloride, which was followed by methanolysis (see Figure 1). Condensation with the corresponding aniline in the presence of *p*-toluene sulfonic acid using a Dean-Stark apparatus and subsequent cyclisation of the enamine at reflux in diphenyl ether gave the corresponding 2-heptyl-4-quinolone. 

**Figure 2:**
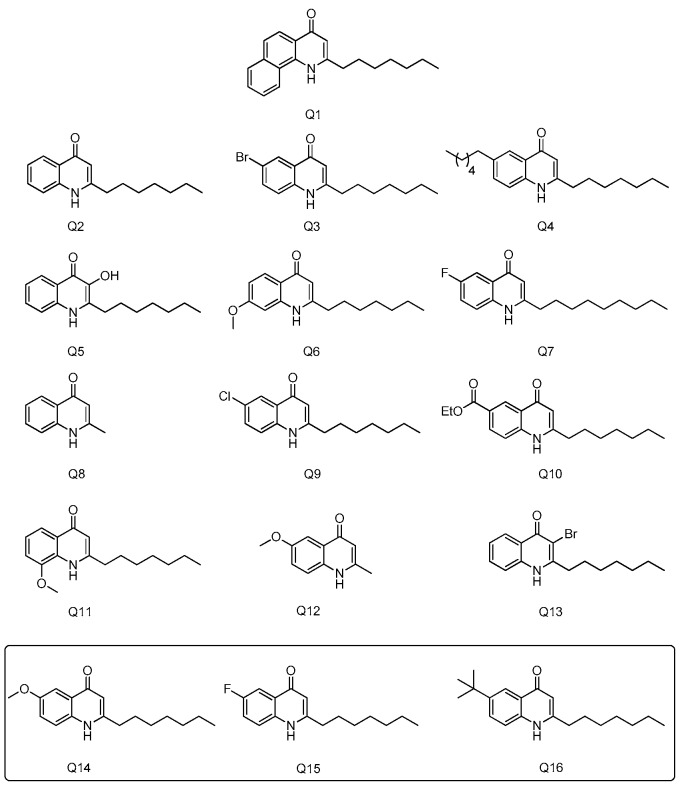
Chemical structure of Quinolones compounds.

Sixteen different quinolones (Q1-Q16) were prepared and used for the investigation of their potential to modulate the GHS-R1a. Substitution of the anthranilate ring (MeO, F, Cl, CO2Et, etc.) focused mainly at the C6 position due to the ease of preparation (only one regioisomer is formed upon cyclisation). However, the 7-OMe and 8-OMe compounds were also prepared and tested. Extension of the aromatic system was also investigated along with the length of the alkyl chain (Me, *n*-heptyl and *n*-nonyl). Lastly, substitution of the C3 position (with a Br) was also tested (see Figure 2).

#### 2.1.2. Quinolones Compounds Activate GHS-R1a-Mediated Intracellular Calcium Mobilization

**Figure 3:**
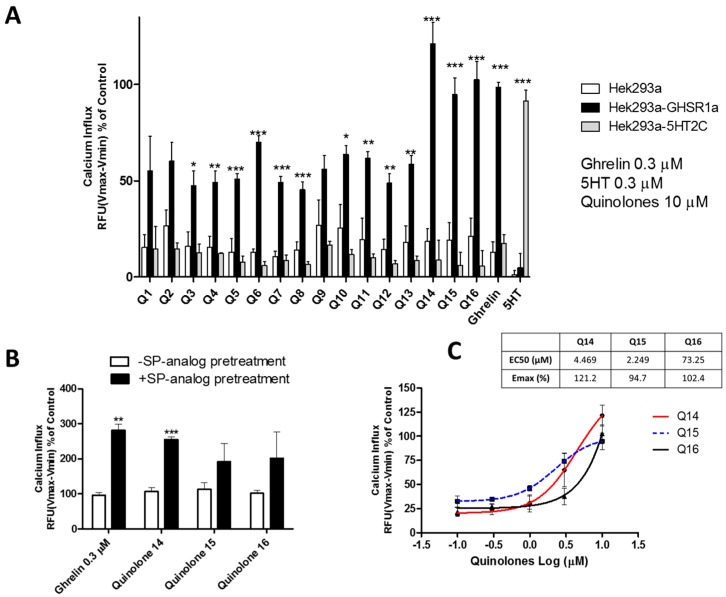
Quinolone compounds (10 µM) specifically activate intracellular calcium influx in Hek-GHS-R1a-EGFP cells. (**A**) Quinolones effects on intracellular calcium influx in wild-type Hek cells. Hek cells stably overexpress the GHS-R1a and Hek cells stably overexpress the 5HT2C receptor. (**B**) Quinolones induced GHS-R1a activation was enhanced following attenuation of constitutive receptor activity by pre-treatment with the GHS-R1a inverse agonist, [D-Arg1, D-Phe5, D-Trp7,9, Leu11]-substance P (1 µM, SP-analogue), (**C**) Quinolones dose-response curve on the intracellular calcium influx in Hek cells stably overexpressing the GHS-R1a. Graphs represent the mean ± SEM from at least four independent experiments with each sample performed in triplicate. The intracellular calcium increase was depicted as a percentage of maximal calcium increase as elicited by the control (3.3% FBS). Significant differences are presented by * indicating *p* ≤ 0.05, ** indicating *p* ≤ 0.01 and *** indicating *p* ≤ 0.001. For further statistic details, see the methods section.

An initial screening involving the diverse set of quinolone compounds was carried out in order to investigate their potency to activate the GHS-R1a by measuring intracellular calcium mobilization [22,40]. Primary signaling of the GHS-R1a is mediated via Gq/_11_-dependent signaling, which leads to the activation of phospholipase C (PLC) and second messengers, inositol trisphosphate (IP_3_) and diacylglycerol (DAG). This leads to increased intracellular calcium influx from the endoplasmic reticulum [41,42]. Exposure to quinolone compounds at 10 µM led to a small intracellular calcium influx (<25%) in wild-type Hek293a cells. An increased calcium influx was observed in Hek cells overexpressing the GHS-R1a (Hek293a-GHS-R1a-EGFP) (see Figure 3A). In addition, this effect was not observed in Hek cells stably overexpressing the serotonin receptor (Hek293a-5HT2C-EGFP), which suggests at least some selectivity of quinolones for the GHS-R1a (see Figure 3A). Although most of the quinolone compounds displayed calcium influx levels below those generated by the endogenous ligand ghrelin (0.3 µM), quinolones Q14, Q15, and Q16 facilitated intracellular calcium mobilization to a similar extent to that exerted by ghrelin and were selected for further investigations (see Figure 3A). Full experimental procedures and characterization of our “hit” quinolones Q14, Q15, and Q16 as well as the ^1^H and ^13^C NMR spectra are included in the Supplementary files. The resazurin assay, which is a widely used method to analyze the viability of bacteria and mammalian cells, demonstrated the non-toxicity of the selected quinolone compounds (see Figure S1) [43]. The Q14, Q15, and Q16 quinolones were also able to mediate intracellular calcium influx following pre-treatment to the inverse agonist [D-Arg1, D-Phe5, D-Trp7,9, Leu11]-substance P (SP-analog) (1 µM) (see Figure 3B). Inverse agonists decrease the constitutive basal activity of G protein-coupled receptors (GPCRs), which reduces its internalization from the membrane to the cytoplasm [44]. In addition, exposure to the SP-analog has been shown to significantly reduce GHS-R1a constitutive activity, which increases its cell surface expression and leads to sensitization of GHS-R1a signaling [45,46,47]. We have also shown multiple times in our laboratory that pre-treatment with the SP-analog leads to a significantly enhanced agonist-mediated calcium mobilization [22,40]. Additionally, SP-analogue pre-treatment significantly enhanced (*p* ≤ 0.01) the endogenous ligand ghrelin-mediated calcium influx in Hek293a-GHS-R1a-EGFP cells (see Figure 3B). A similar effect (*p* ≤ 0.001) was observed for quinolone Q14-mediated response, which indicates high selectivity of this compound for the GHS-R1a (see Figure 3B). Exposure to quinolones Q15 and Q16 also showed an increased intracellular calcium mobilization upon pre-treatment with the SP-analogue but failed to reach significance (see Figure 3B). These quinolone compounds also stimulated an intracellular calcium influx in a concentration-dependent manner with an efficacy of 121.2%, 94.7%, and 102.4% and EC_50_ of 4.5 µM, 2.2 µM, and 73.2 µM for Q14, Q15, and Q16, respectively (see Figure 3C). Furthermore, the most active quinolone Q15 displayed an EC_50_, which was 25-fold higher than that reported for the endogenous GHS-R1a agonist ghrelin (EC_50_ 88 nM) and 314-fold higher when compared to the non-peptide agonist MK-0677 (EC_50_ 7 nM). This highlights the very promising potential of the quinolone molecular template for the development of GHS-R1a targeting therapeutics [22].

**Figure 4:**
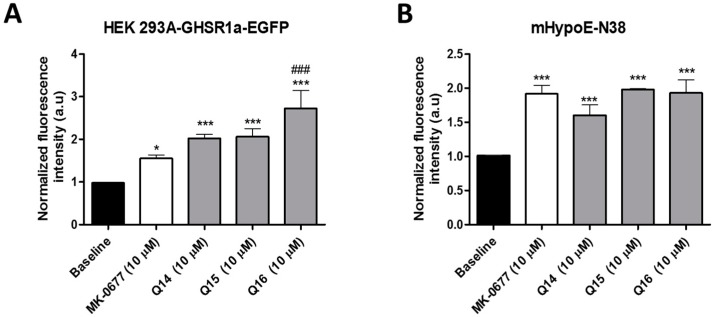
Quinolone compounds activate GHS-R1a receptor overexpressed in Hek cells as well as the endogenous receptor expressed in hypothalamic neurons. Quinolone compounds (10 µM) and MK-0677 activate GHS-R1a receptors in transfected human embryonic kidney (Hek293a) cells (**A**) and embryonic mouse hypothalamic cell line-N38 (**B**) by using cell calcium imaging through confocal microscopy (for visualization, see Supplementary videos in Supplementary files). Significant differences compared to the baseline and MK-0677 controls are presented with * and # respectively with * indicating *p* ≤ 0.05 and ***/### indicating *p* ≤ 0.001. For further statistic details, see the methods section.

Intracellular calcium mobilization was also investigated with calcium imaging using confocal fluorescent microscopy. Exposure to quinolone Q14, Q15, and Q16 led to a significant activation (*p* ≤ 0.001) of calcium mobilization in both Hek cells overexpressing the GHS-R1a as well as in a hypothalamic neuronal cell line model of an embryonic mouse. The hypothalamus cell line-N38 (mHypoE-N38), which possesses endogenous levels of GHS-R1a expression [22] (see Figure 4 and live imaging video clip in Supplementary files). Therefore, the addition of these quinolone compounds showed an increased fluorescence intensity when compared to baseline with similar efficacy as the positive control MK-0677, which is an established non-peptide agonist of the GHS-R1a [48]. In the case of Hek293a-GHS-R1a-EGFP cells, exposure to the quinolones actually resulted in a higher intracellular calcium influx when compared to the GHS-R1a agonist MK-0677 (see Figure 4A). Furthermore, while Q14 and Q15 showed a trend to mediate higher calcium influx (*p* = 0.075 and *p* = 0.085, respectively), Q16 exhibited a significant (*p* ≤ 0.001) higher calcium mobilization when compared to MK-0677 (see Figure 4A). 

**Figure 5:**
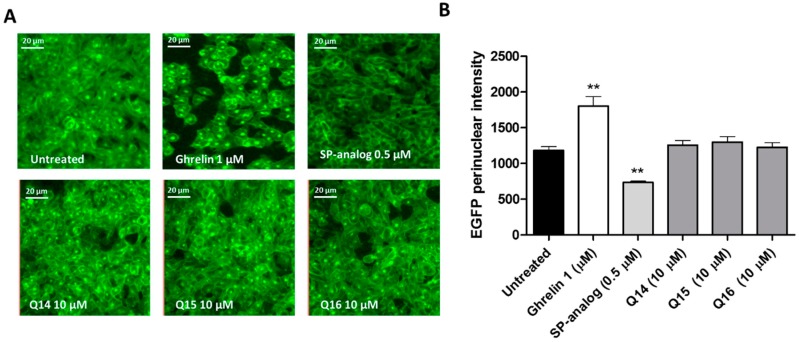
Quinolone compounds’ effects on the GSH-R1a internalization. Quinolone compounds do not affect GSH-R1a internalization. (**A**) Representative images are depicted following different treatments: the untreated control (assay buffer), ghrelin (1 μM), [D-Arg1, D-Phe5, D-Trp7,9, Leu11]-substance P (SP-analog) (0.5 μM) and quinolones Q14, Q15 and Q16 (10 μM). (**B**) The graph represents the mean ± SEM of quantified fluorescence intensity (15 pictures per treatment) of the perinuclear GHS-R1a-EGFP receptor from four independent experiments with each treatment performed in triplicates. ** indicates *p* ≤ 0.01. For statistic details, see the methods section.

Finally, the effect of the quinolones on GHS-R1a internalization was investigated in Hek cells by stably overexpressing the GHS-R1a-EGFP. Receptor trafficking from the membrane to the cytoplasm was analyzed by quantifying the EGFP fluorescence intensity. As expected, exposure to the endogenous ligand ghrelin (1 µM) led to significantly (*p* ≤ 0.01) increased GHS-R1a internalization while exposure to the inverse agonist SP-analog led to a significant (*p* ≤ 0.01) opposite effect when compared to untreated cells (see Figure 5). However, exposure to the quinolones compounds did not show any effect on the GHS-R1a internalization process (see Figure 5).

## 3. Discussion

Cachexia affects between 10% and 40% of patients with chronic diseases and there is no effective therapy currently available [49]. One new approach to treat this complex metabolic syndrome is focused on the orexigenic peptide ghrelin, which increases appetite and growth hormone secretion [50]. Indeed, several beneficial effects of ghrelin in the treatment of cachexia have been reported, such as increased energy consumption, increased appetite and muscle tissue, reduction of adipose tissue loss, and improved respiratory muscle strength (see Reference [50]). However, due to the necessity of parenteral administration, ghrelin’s short half-life, and potential side-effects, its efficacy in patients is restricted [20]. Therefore, the development of new cachexia therapeutics with the ability to mimic ghrelin actions but with improved efficacy in patients is necessary. Quinolone compounds are mainly known for their widespread antimicrobial properties [51] but they have also been shown to exert different beneficial effects including antiulcer, antihistamine, or antimutagenic properties [34]. Recently, quinolones have been studied as signaling molecules for the interruption of quorum sensing in *Pseudomonas aeruginosa* and other pathogens [35]. Quinolones are very versatile structures that can be easily modified to enhance their interaction with key active sites. Different functional groups can be incorporated to 4-(1*H*)-quinolones on the anthranilate backbone or at the C-2 position via modification of the ester precursor [52]. Late stage alteration (e.g., halogenation) of the key C-3 position can also be achieved to further fine-tune the structure-activity relationship [53]. Following on from our previous study on the use of 2-pyridones [22], we investigated the potential of quinolone compounds synthesized in our lab to modulate appetite in our well-established screening platform by using Hek cells stably overexpressing GHS-R1a, which is the receptor of the hunger hormone ghrelin. 

A number of quinolones (Q14, Q15, Q16) displayed intracellular calcium mobilization to a similar extent to that exerted by ghrelin in Hek cells stably overexpressing the GHS-R1a. This indicates a significant potential to modulate the Gq/_11_-dependent GHS-R1a signaling pathway (see Figure 3A). Exposure to quinolones did not lead to increased calcium influx in Hek293-WT cells. The quinolones screened in this study have a long hydrophobic heptyl chain at the C2 position, which, taken together with the more polar quinolone part of the molecule, can grant unique lipid-type qualities. Interestingly, a longer alkyl chain at C2 has proven critical for biological activity in a number of diverse settings [33]. In this research study, the presence of the heptyl chain at the C2 position appeared to play an important role also. For example, analog Q14 showed good intracellular calcium mobilization while Q12, which differs only at C2 and possesses a shorter alkyl chain, did not. Remarkably, Q7 was not very active and differed only by two carbon units from the active Q15. That said, not all quinolones with a C2 heptyl chain were highly active. The presence of substituents at the C6 position appeared to be well tolerated, which allowed for secondary modification. However, not all substitution at C6 gave quinolones capable of efficient calcium mobilization. Quinolones with electron-donating groups (Q14 and Q16) were active, but those with electron withdrawing groups (Q9, Q10) were generally not with the exception of Q15. Overall, remarkable structural specificity was observed but no clear trend could be identified, perhaps highlighting the specific requirements for GHS-R1a activation. 

The three most active quinolones Q14, Q15, and Q16 were selected for further investigations. An increased calcium influx was also observed following exposure to these quinolones in hypothalamic neurons (see Figure 4) with endogenous levels of GHS-R1a expression, which suggests their potential to activate hypothalamic neurons [22]. The GHS-R1a has a very high ligand-independent signaling or constitutive signaling at approximately 50% of its maximal Gq/11-mediated efficacy [46]. The high constitutive GHS-R1a activity serves as a desensitization mechanism, which internalizes the receptor after signaling into endosomes and sequesters the receptor from the membrane to the cytoplasm in an endocytosis process mediated by β-arrestin [46]. Inverse agonists have been shown to reduce receptor constitutive activity and to increase receptor membrane expression and enhance ligand-mediated calcium signaling [45,47]. Additionally, Q14 showed an enhanced effect on calcium signaling upon pre-treatment with an SP-analog as well as an ability to activate the GHS-R1a in a concentration-dependent manner (see Figure 3B,C), which indicates the high specificity of this compound for the GHS-R1a. Q15 and Q16 also showed an increased calcium influx following SP-analog pre-treatment but they failed to be significant. In previous studies from our laboratory, we have also shown that the basal levels of GHS-R1a constitutive signaling decrease upon exposure to the inverse agonist SP-analog [22,40]. However, while quinolones elicited a significant GHS-R1a-mediated intracellular calcium mobilization, no GHS-R1a internalization was observed (see Figure 5), which suggests that these quinolone compounds may act as functional selective or biased GHS-R1a agonists. These compounds activate the Gαq/11-mediated pathway but are unable to trigger β-arrestin recruitment and subsequent GHS-R1a receptor internalization and desensitization [54,55,56]. An example of another ghrelin receptor ligand known as JMV3018 was also able to partially activate the Gαq-protein signaling pathway but unable to recruit β-Arrestin [57]. In addition, recent studies including a study carried out in our laboratory [58] have demonstrated that GHS-R1a receptor ligands display signaling bias with functional selectivity for specific downstream signaling pathways [22,59,60,61,62]. Indeed, several different active conformations of the GHS-R1a receptor may exist [42], with biased ligands able to induce a receptor conformation that preferentially activates only G protein-mediated or β-arrestin-mediated downstream signaling [63]. 

This biased modulation of GPCRs is becoming increasingly recognized in GPCR-drug discovery, which may allow for the discovery of more selective therapeutic compounds meant for the most appropriate GPCRs mediated functionalities [63]. Taken together, we provide compelling evidence for quinolone-mediated GHS-R1a activation with functional selectivity for the Gq/11 pathway. Further studies are needed to fully understand the role of this functional selectivity in mediating the effects of GHS-R1a receptor ligands. 

In conclusion, we have identified non-peptide quinolone compounds with promising GHS-R1a activating potential in vitro. In terms of a structure activity-relationship, the results described herein point to the necessity of a C2 heptyl chain (a shorter Me group leads to compounds displaying a decrease in calcium intake (see Figure 1A)). Furthermore, a comparison of the hit compound Q15 possesses a C2-heptyl chain with others such as Q7, which possesses a C2-nonyl chain. This indicates the remarkable requirements of the chain length. Direct comparison of substitution at C6 and C7 (Q11 and Q14) shows the benefit of C6 substitution. Additionally, electron withdrawing groups (Q15) and electron donating (Q14) are equally tolerated at C6, which as the bulky *t*-Bu group (Q16). Such disregard for the electronics and sterics at C6 is in stark contrast to the exquisite specificity required of the chain length at C2. 

It will be interesting to see how additional variants of the quinolone scaffold modulates the GHS-R1a receptor in vitro and how this translates to food intake and anabolic effects in vivo. The focus will be placed on the development of quinolones with substitution at C6 in concert with fine-tuning further hits. Certainly, further development of the quinolone molecular template is warranted to explore its therapeutic potential in metabolic disorders including cachexia and obesity via the modulation of the GHS-R1a receptor. 

## 4. Materials and Methods

### 4.1. General Procedure for the Preparation of Substituted 2-Alkyl-4-Quinolones

#### 4.1.1. Preparation of the β-Ketoester

2,2-Dimethyl-1,3-dioxane-4,6-dione (Meldrum’s acid) (18.7 g, 130 mmol) was dissolved in distilled dichloromethane (200 mL). The solution was cooled to 0 °C under N_2_ atmosphere. Pyridine (20.5 mL, 260 mmol) and octanoyl chloride (23.8 mL, 140 mmol) were added to the cooled solution, dropwise. The solution was stirred at 0 °C for 1 h and then, at room temperature, for 1 h. The mixture was washed with 5% HCl (3 × 75 mL) and with distilled water (75 mL). The solution was then dried with anhydrous MgSO_4_ filtered and concentrated in vacuum to yield acyl Meldrum’s acid as a brown oil, which was used in the subsequent step without further purification.

Acyl Meldrum’s acid was dissolved in MeOH (180 mL) and heated at reflux for 5 h with constant stirring. After allowing to cool, the reaction mixture was concentrated in vacuum yielding the crude product as an orange oil. Purification was achieved by fractional distillation, which affords the β-keto ester as a pale yellow oil (16.7 g, 64% yield). The analytical data was consistent with previously published studies [64].

#### 4.1.2. Preparation of the Quinolones

To a solution of the β-ketoester (5 mmol) in hexane (10 mL) were added to the corresponding aniline (5 mmol) and *p*-toluene sulfonic acid (0.1 mmol). The reaction mixture was heated at reflux (>70 °C) under N_2_ atmosphere overnight using a Dean-Stark system. Upon completion, the reaction mixture was concentrated in vacuum to afford the crude β-enamino ester, which was then added drop-wise to refluxing diphenyl ether (2 mL, >260 °C). Reflux was maintained for approximately 1.5 h. After cooling to room temperature, ether (approximately 20 mL) was added to the reaction mixture and left overnight at 5 °C, which allows for the quinolone product to precipitate. The quinolone was collected by vacuum filtration, recrystallized from hot methanol (if necessary), and dried under vacuum.

Solvents and reagents were used as obtained from commercial sources and used without purification. ^1^H NMR (300 MHz) spectra were recorded on Bruker Avance 300 NMR spectrometers in proton-coupled mode. ^13^C NMR (75.5 MHz) spectra were recorded on Bruker Avance 300 NMR spectrometers in proton decoupled mode at 20 °C in deuterated dimethyl sulphoxide or CDCl_3_ using tetramethylsilane as an internal standard. High-resolution precise mass spectra (HRMS) were recorded on a Waters LCT Premier Tof LC-MS instrument in electrospray ionization (ESI) mode using 50% acetonitrile-water containing 0.1% formic acid as the eluent. Samples were made up in acetonitrile. Infrared spectra were measured as pressed potassium bromide (KBr) for solids or thin films on sodium chloride plates for liquids on a Perkin-Elmer FT-IR spectrometer.

### 4.2. Compounds Preparation

All compounds including the ghrelin receptor agonists ghrelin (Innovagen, Lund, Sweden) and MK-0677 (Tocris, R&D Systems, Bristol, UK) as well as the inverse agonist [D-Arg1, D-102 Phe5, D-Trp7,9, Leu11] Substance P (SP-analogue) (Tocris, R&D Systems, Abingdon, UK) and the serotonin receptor agonist 5-hydroxytryptamine (5-HT, H9523; Sigma, St. Louis, MO, USA) were prepared in assay buffer (1× Hanks balanced salt solution, HBSS, (Gibco, Waltham, MA, USA), which contained 20 mM HEPES (Sigma-Aldrich, St. Louis, MO, USA)).

### 4.3. Cell Culture

Human embryonic kidney cells (Hek293a) (Invitrogen, Carlsbad, CA, USA) were cultured in high glucose Dulbecco’s modified Eagle’s medium (DMEM, Invitrogen) containing 10% heat-inactivated fetal bovine serum (FBS) (Sigma-Aldrich) and 1% non-essential amino acids (NEAA) (Gibco) at culture conditions (37 °C and 5% CO_2_ in a humidified atmosphere). Hek293a cells were transfected with a plasmid construct expressing the human GHS-R1a receptor with a C-terminal enhanced green fluorescent protein (EGFP) tag as previously described [31] and cultured in complete DMEM media, which contains 300 ng/µL G418 (Calbiochem, Merck KGaA, Darmstadt, Germany) as maintenance antibiotic. 

Embryonic mouse hypothalamus cell line N38 (mHypoE-N38) (Cederlane Laboratories, Cellutions Biosystems, Burlington, ON, Canada) was maintained in high glucose Dulbecco’s modified Eagle’s medium (DMEM, Invitrogen) containing 10% heat-inactivated fetal bovine serum (FBS) (Sigma-Aldrich) at culture conditions (37 °C and 5% CO_2_ in a humidified atmosphere).

Cells were grown to a confluence of >85% and subsequently split to a lower density for continued culturing. 

### 4.4. Calcium Mobilization Assay

G-protein coupled receptor-mediated changes in intracellular calcium (Ca^2+^) were investigated using a Flex station II multiplate fluorometer (Molecular Devices Corporation, Sunnyvale, CA, USA) based on protocols described in previous studies [22,31,40]. Briefly, wild-type Hek293a cells stably expressing the GHS-R1a or the 5HT2C receptors were seeded in black 96-well microtiter plates (2.8 × 10^4^ cells/well) and maintained for about 24 h at cultured conditions. Growth media was then replaced by serum-free DMEM media containing 1% NEAA and the cells were incubated for another 24 h at cultured conditions. Next, cells were incubated for 90 min with assay buffer (1× Hanks balanced salt solution, HBSS, containing 20 mM HEPES) and 1x Ca4 dye (Molecular Devices Corporation) (1:1 *v/v*), according to the manufacturer’s instructions. The quinolones compounds were dissolved in assay buffer at 10 µM containing 0.1% DMSO (Sigma-Aldrich). Finally, compounds were automatically added by the Flexstation II during continuous fluorescent measurements for a total of 80 s at 37 °C in flex mode with an excitation wavelength of 485 nm and an emission wavelength of 525 nm. The relative increase in intracellular calcium [Ca^2+^] was calculated as the difference between maximum and baseline fluorescence (Vmax-Vmin) and depicted as a percentage of relative fluorescent units (RFU), which is normalized to a maximum response (100% signal) obtained with 3.3% FBS. Background fluorescence was obtained by cells in assay buffer alone and subtracted from RFUs. Exposure to quinolones following a pretreatment to the inverse agonist [D-Arg1, D-Phe5, D-Trp7,9, Leu11]-substance P was also carried out. This pretreatment was performed during the calcium dye incubation. Data were analyzed using the GraphPad Prism software (PRISM 5.0; GraphPAD Software Inc., San Diego, CA, USA). Sigmoidal dose-response curves were constructed using nonlinear regression analysis with a variable slope excluding values resulting from obvious incorrect pipetting by the Flexstation II (Molecular Devices Corporation).

### 4.5. Calcium Imaging

Calcium imaging experiments were carried out as previously described [22]. Hek-GHSR-1a-EGFP and mHypoE-N38 cells were seeded in poly-l-lysine coated 35 mm dishes (MatTek Corporation, Ashland, MA, USA) at a density of 15 × 104 cells/ml and maintained for ~24 h at culture conditions (37 °C and 5% CO_2_ in a humidified atmosphere). After 24 h, cells were incubated for 40 min at 37 °C in Fluo-4 solution (Molecular probes Incorporation., Fisher Scientific, Waltham, MA, USA)) at 4 µM or 8 µM for Hek-GHS-R1a-EGFP and mHypoE-N38 cells, respectively. After removal of the dye, cells were washed twice with PBS and 2 mL of assay buffer (1× Hanks balanced salt solution, HBSS, containing 20 mM HEPES) were added to the cells, which were stored in the dark at 37 °C until visualization under the microscope. 

Each dish plate was mounted on the stage under culture conditions (37 °C and 5% CO_2_ in a humidified atmosphere) for stable recording for a longer period of time using laser scanning confocal fluorescent microscopy (FV 1000 Confocal System; Olympus, Tokyo, Japan). Fluorescence was visualized on an inverted microscope (CKX41; Olympus) setup with a sensitive XM10 camera (C-BUN-F-XM10-BUNDLE) with an infrared cut filter, mercury burner (USH-103OL, Ushio America, Cypress, CA, USA), and fluorescence condenser (CKX-RFA; Olympus). Green Fluorescence of tagged GFP protein was monitored with an excitation wavelength of 485 nm and an emission wavelength of 525 nm. The same settings were used to monitor calcium mobilization signals. Image acquisition was carried out at 500 µs/pixel. Cells within a single field of view were imaged over a 1 min period in the absence of the compound (line base) with a 10-s shuttered interval between each image. When each compound was added, the field of view was imaged over a 2 min period with a 1-s shuttered interval between each image.

### 4.6. Internalization Assay

Receptor translocation from the membrane to the cytoplasm was carried out as previously described [22]. Hek-GHSR1a-EGFP cells were seeded in a poly-l-lysine (Sigma-Aldrich) coated 96-well microtiter plate at 3 × 10^4^ cells per well and incubated for 48 h at cultured conditions. For the last 24 h of this time period, media was replaced with serum-free DMEM media. Cells were exposed to quinolone compounds at 10 µM containing 0.1% DMSO for 1 h at 37 °C. Ghrelin was used as a positive control. In addition, treatment with the inverse agonist, [D-Arg1, D-Phe5, D-Trp7,9, Leu11]-substance P, was also carried out. The cells were then fixed with 4% paraformaldehyde in phosphate buffer saline (PBS) for 20 min, washed three times with PBS, and imaged on the GE Healthcare IN Cell Analyser 1000 (GE Healthcare, Buckinghamshire, UK) in PBS. Receptor GHS-R1a-EGFP trafficking was quantified by analyzing the EGFP fluorescence perinuclear intensity using the INCell Analyzer Developer Toolbox V1.6 software (GE Healthcare) as previously described [22]. Data were analyzed and depicted using GraphPad Prism software (PRISM 5.0; GraphPAD Software Inc., La Jolla, CA, USA).

### 4.7. Statistical Analysis

Statistical analysis was performed using SPSS software (IBM SPSS statistics version 22, Armonk, NY, USA). Normality of the data was tested by using the Shapiro-Wilk test. Normally distributed data were analyzed by one-way ANOVA followed by the Dunnett test (when comparing against a control group) or by the Student’s *t*-test (when comparing 2 groups, see Figure 3B). A non-parametric multiple comparisons Kruskal-Wallis test was performed if data was not normal, which was followed by, where appropriate, Mann-Whitney U tests for individual comparisons. The level of significance in all analysis was α = 0.05 and all tests were a two-tailed test. All graphs represent the mean ± SEM from N independent experiments (see figure legends for N details for each experiment). Statistical significances are subsequently depicted as follows: * indicating *p* ≤ 0.05, ** indicating *p* ≤ 0.01 or *** indicating *p* ≤ 0.001.

## Supplementary Materials

Supplementary materials can be found at http://www.mdpi.com/1422-0067/19/6/1605/s1.
